# Crohn's Disease and Urinary Bladder Mass

**DOI:** 10.1155/DTE.1.233

**Published:** 1995

**Authors:** Gregory T. Bales, Francis H. Straus, II, Glenn S. Gerber

**Affiliations:** 1 Section of Urology Department of Surgery and Department of Pathology University of Chicago Pritzker School of Medicine Chicago Illinois USA; 2 University of Chicago Hospitals 5841 S. Maryland Avenue Section of Urology MC 6038 Chicago IL 60637 USA

## Abstract

The presence of a bladder mass in a patient with inflammatory bowel disease poses a diagnostic dilemma. We present the case of a 26-year-old male with a bladder mass who had not previously been diagnosed with Crohn's disease. Initial biopsies of the bladder mass were consistent with inflammatory changes, but superficial transitional cell carcinoma could not be reliably excluded. Subsequent evaluation confirmed the presence of Crohn's disease with bladder involvement, and the patient underwent bowel resection and partial cystectomy. Pathologic evaluation demonstrated Crohn’s disease and no evidence of malignancy. Accurate differentiation of benign and malignant bladder masses in patients with inflammatory bowel disease may be difficult and requires cooperation between pathologists and clinicians.


*Diagnostic and Therapeutic Endoscopy*, 1995, Vol. 1, pp. 233–236
Reprints available directly from the publisher
Photocopying permitted by license only

© 1995 Harwood Academic Publishers GmbH
Printed in Singapore

### Crohn's Disease and Urinary Bladder Mass

GREGORY T. BALES, FRANCIS H. STRAUS, II, and GLENN S. GERBER

*Section of Urology, Department of Surgery and Department of Pathology, University of Chicago Pritzker School of Medicine, Chicago, Illinois, USA*

*(Received September 9, 1994; in final form December 9, 1994)*

The presence of a bladder mass in a patient with inflammatory bowel disease poses a diagnostic dilemma. We present the case of a 26-year-old male with a bladder mass who had not previously been diagnosed with Crohn's disease. Initial biopsies of the bladder mass were consistent with inflammatory changes, but superficial transitional cell carcinoma could not be reliably excluded. Subsequent evaluation confirmed the presence of Crohn's disease with bladder involvement, and the patient underwent bowel resection and partial cystectomy. Pathologic evaluation demonstrated Crohn's disease and no evidence of malignancy. Accurate differentiation of benign and malignant bladder masses in patients with inflammatory bowel disease may be difficult and requires cooperation between pathologists and clinicians.

**KEY WORDS:** Crohn's disease, bladder mass, inflammatory bowel disease

The association between inflammatory bowel disease and an increased risk of extraintestinal cancers has been well established (1). In particular, Nakajima, et al. and Greenstein, et al. have demonstrated that genitourinary malignancies are more commonly seen in patients with Crohn's disease (2,3). These authors and others also have reported that transitional cell carcinoma of the urinary bladder may occur with increased frequency in patients with Crohn's disease and that these tumors may occur at a much younger age than is usually seen (4). Conversely, there have been rare reports of Crohn's disease mimicking a bladder tumor (5–7). Therefore, the presence of a bladder mass in a young person with inflammatory bowel disease often presents a diagnostic dilemma.

### CASE REPORT

A 26-year-old Greek male was referred for evaluation of an anterior bladder mass that had been noted on cystoscopy. The patient had experienced lower abdominal discomfort and dysuria for several months leading to a urologic evaluation. Computerized tomographic scans demonstrated the mass in the bladder with no evidence of extravesical extension or inflammation (Fig. 1). Transurethal biopsy of the mass was performed, which demonstrated a hyperplastic transitional cell layer with a convoluted mucosal surface and extensive acute and chronic granulomatous cystitis, cystitis cystica, and cystitis glandularis (Fig. 2). The specimen displayed an irregular surface with broad fibrovascular cores between thickened layers of transitional cells that was consistent with a low-grade, superficial transitional cell carcinoma. Subsequently, the patient developed recurrent abdominal discomfort as well as pneumaturia. A cystogram demonstrated an enterovesical fistula, and radiographic studies of the gastrointestinal tract revealed irregular clefts in the small and large bowel with severe stenotic changes consistent with Crohn's disease. The patient underwent laparotomy with bowel resection and partial cystectomy. Histologic review demonstrated follicular cystitis and findings characteristic of Crohn's disease in the cecum. Therefore, it was concluded that the bladder mass and pathologic findings noted on transurethral biopsy were secondary to Crohn's disease and not transitional cell carcinoma. Pathologically, it was impossible to ascertain if

Address for correspondence: Glenn S. Gerber, MD, University of Chicago Hospitals, 5841 S. Maryland Avenue, Section of Urology, MC 6038 Chicago, IL 60637

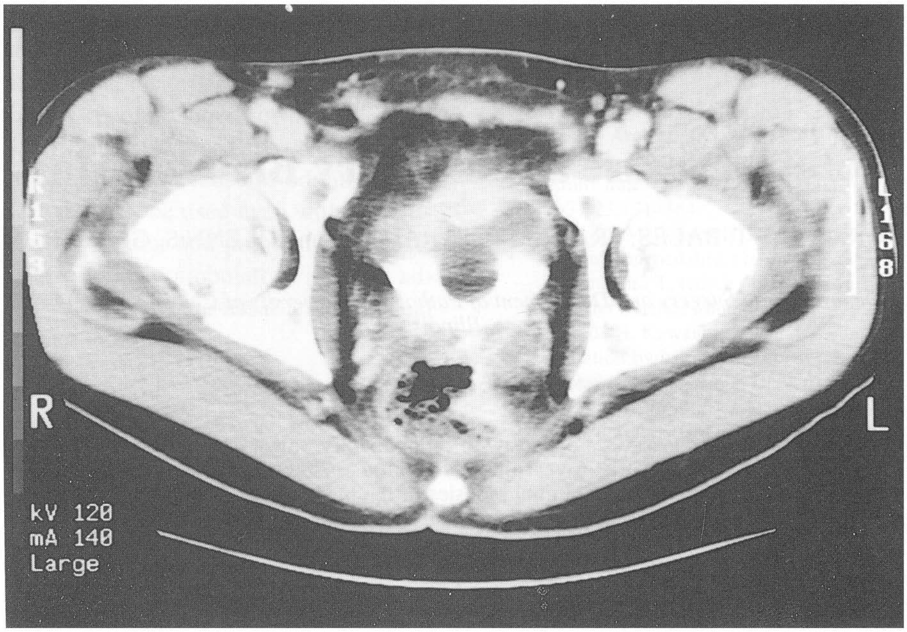

**Figure 1** Computerized tomographic image demonstrating mass in bladder.

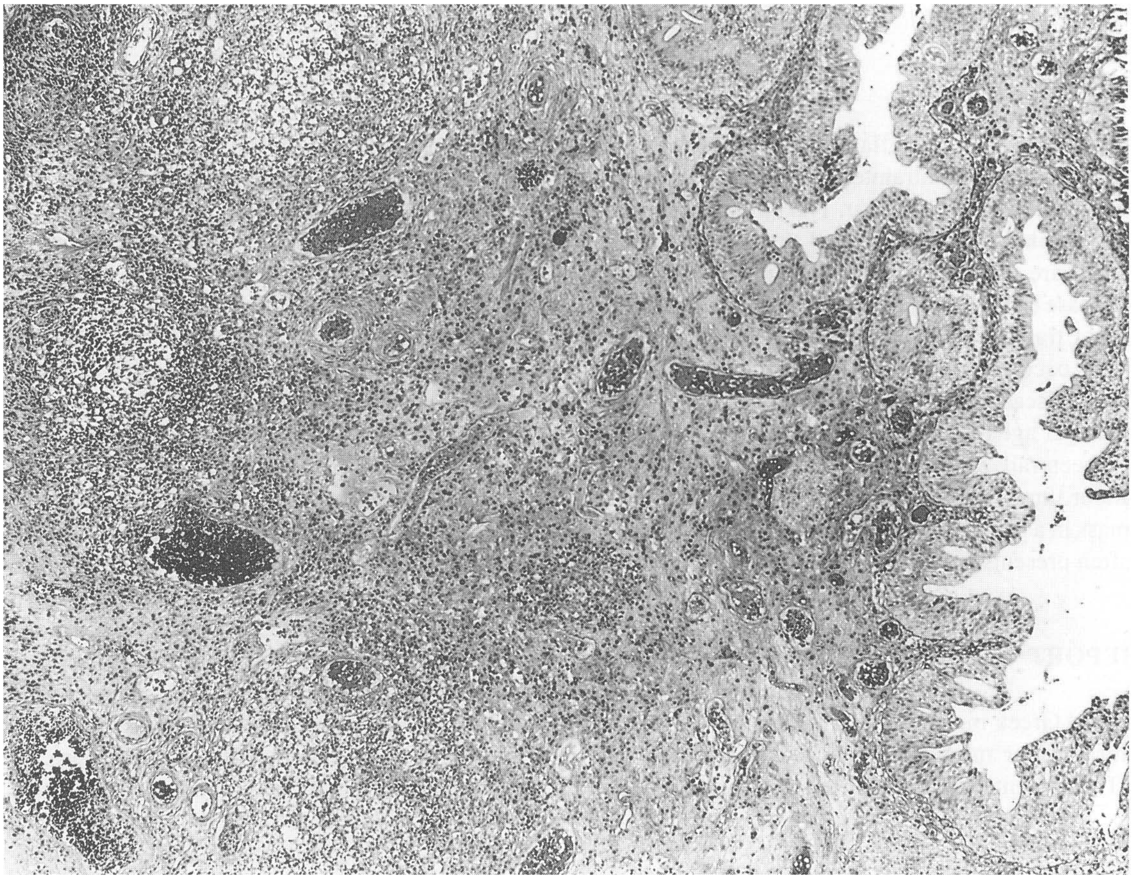

**Figure 2a** Dense inflammatory infiltrate in bladder wall with cystitis clystica and cystitis glandularis.

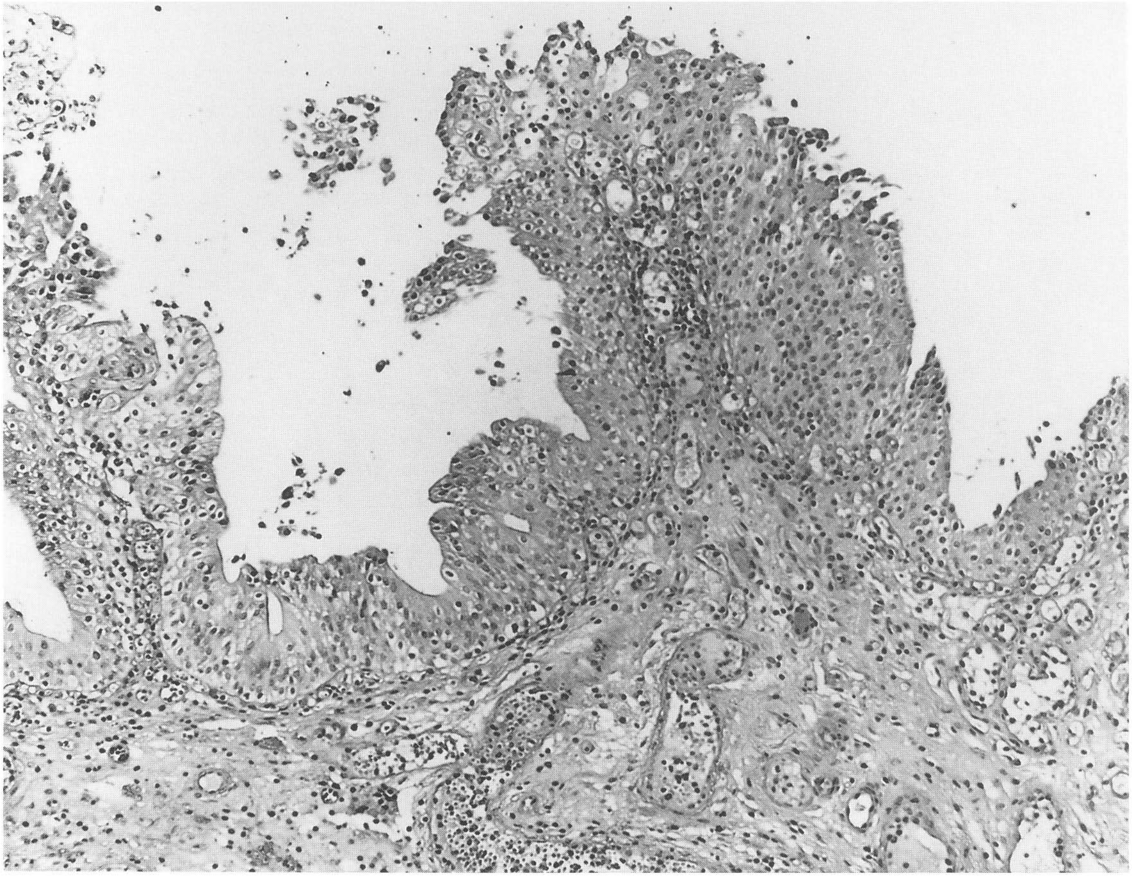

**Figure 2b** Hyperplastic transitional cell layer with convoluted mucosal surface.

this patient had primary involvement of the bladder by Crohn's disease or if the mass arose secondary to adjacent bowel inflammation. After surgery, the patient had an uneventful recovery with resolution of his symptoms.

### DISCUSSION

The radiographic and endoscopic demonstration of a mass in the urinary bladder most commonly represents a transitional cell carcinoma. However, Crohn's disease may involve the urinary tract in a benign fashion and appear as a mass in the bladder caused by compression and invasion by an enlarging intraabdominal abscess (5,7). In addition, enterovesical fistulas may develop secondary to regional enteritis and lead to an appearance consistent with a bladder tumor (6). Finally, the bladder may become segmentally involved with Crohn's disease. Although it has been previously demonstrated that urinary bladder masses may occur secondary to Crohn's disease, it also has been shown that young patients with inflammatory bowel disease have an increased risk of transitional cell carcinoma of the bladder (2–4).

This case demonstrates the difficulties in distinguishing primary bladder tumors from benign inflammatory masses in those with Crohn's disease. Physicians should consider the inflammatory manifestations of regional enteritis when confronted with a young patient with a bladder mass who has no apparent risk factors for transitional cell carcinoma of the urinary tract. Conversely, the increased incidence of genitourinary tumors found in patients with inflammatory bowel disease also must be considered, and the possibility of malignancy should not be dismissed without careful analysis. Pathologic interpretation of biopsy material in these cases often is problematic, and the distinction between low-grade cancers and severe inflammatory changes may be difficult. It is essential that clinicians and pathologists work closely together to allow for accurate differentiation between benign and malignant disease.

### REFERENCES

1. Greenstein AJ, Sachar DB. Cancer in inflammatory bowel disease. Surv Dig Dis 1983;1:8–18.
2. Nakajima H, Munakata A, Yoshida Y. Extraintestinal cancers in Crohn's disease. Digestion 1990;47:1–7.
3. Greenstein AJ, Gennuso R, Sachar DB, et al. Extraintestinal cancers in inflammatory bowel disease. Cancer 1985;56:2914–2921.
4. Fujimjura Y, Kihara T, Uchida J, et al. Transitional cell carcinoma of the bladder associated with Crohn's disease: case report and review of the literature. Brit J Radiol 1992;65:1040–1042.
5. Evans RH. Crohn's disease mimicking primary bladder tumour. Brit J Urol 1990;65:299–300.
6. Yamamoto M, Ando T, Kanai S, et al. Enterovesical fistula due to Crohn's disease masquerading as bladder tumor. Acta Urol Jpn 1986;32:1141–1143.
7. Goldstein MJ, Bragg D, Sherlock P. Granulomatous bowel disease presenting as a bladder tumor: report of a case. Am J Dig Diseases 1971;16:337–341.

